# Heterogeneity of type 2 diabetes in rural India

**DOI:** 10.3389/fendo.2025.1524194

**Published:** 2025-09-09

**Authors:** Suvarna Patil, Ajay Patil, Kaustubh Tare, Pallavi Bhat, Akash Kumbhar, Datta Mulay, Dnyaneshwar Jadhav, Vaibhav Methi, Sachin Surnar, Tushar Humbare, Aniket Khaladkar, Shreyansh Deosale, Charudatta Joglekar

**Affiliations:** ^1^ Department of Medicine, BKL Walawalkar Rural Medical College and Hospital, Sawarde, Chiplun, Maharashtra, India; ^2^ Regional Centre for Adolescent Health and Nutrition, BKL Walawalkar Rural Medical College and Hospital, Sawarde, Chiplun, Maharashtra, India

**Keywords:** type 2 diabetes, India, rural health, body composition, C-peptide, non-obese, lean diabetes

## Abstract

**Background:**

The prevalence of diabetes continues to rise in India. The increase in diabetes, which has been previously concentrated in urban areas, is now also occurring in rural India, even though the rural population is predominantly non-obese/lean, undernourished, and physically active. Type 2 diabetes and its pathophysiology (hyperinsulinemia and insulin resistance) among the obese, overnourished, and physically inactive urban populations are very well characterized. However, there is a paucity of such characterization among those non-obese/lean and undernourished. We attempted to characterize type 2 diabetes in the rural Konkan region of India using BMI, body composition, and glycemic parameters.

**Methods:**

This cross-sectional study was conducted among 508 subjects with type 2 diabetes who visited the rural tertiary care center. They underwent anthropometry, body composition, and glycemic (i.e., glucose, HbA_1C_, and C-peptide) measurements. Various homeostatic model assessment (HOMA) indices for insulin resistance (HOMA-IR), β-cell function (HOMA-β), and insulin sensitivity (HOMA-S) were calculated. The subjects were classified into six groups, with the classification system created by combining BMI with the parameters of central obesity and adiposity for comparisons of the glycemic measurements and the HOMA indices.

**Results:**

The median age, the age at diagnosis, and the duration of type 2 diabetes were 59, 51.5, and 5.2 years, respectively. The BMI distribution showed that 6% were underweight, approximately 46% were normal weight, and 48% were overweight. Central obesity and adiposity were observed in 70% and 56%, respectively. Of those subjects with normal weight, 52% had central obesity, while 29% had excess adipose tissue. The lean group was characterized by a low BMI (mean, 16.7 kg/m^2^), the absence of central obesity and adiposity, and the lowest C-peptide (median, 463.5 pmol/L) and the lowest insulin resistance. The glycemic characteristics of the overweight, centrally obese, and adipose subjects were similar to those observed in classical type 2 diabetes.

**Conclusion:**

The use of BMI and simple body composition measures led to the identification of a distinct lean phenotype that is characterized by a low BMI, poor insulin secretion, and the absence of central obesity and adiposity. Further research is warranted to understand the pathophysiology and to develop a personalized therapeutic approach for lean subjects with type 2 diabetes. It is time to reconsider the glucocentric, one-size-fits-all approach for type 2 diabetes treatment.

## Introduction

Type 2 diabetes mellitus (T2DM) is on the rise globally, and India has become its epicenter. India has the second largest population (79 million) of people with T2DM, which is expected to reach 145 million by 2045 (1, 2). Diabetes now affects large parts of both urban and rural India (3). Obesity [usually associated with a high body mass index (BMI)], physical inactivity, and an unhealthy diet have been identified as the major contributing factors. However, the contribution of the non-obese populations to the total burden of T2DM has been reported to be 10%–20%, but is as high as 60%–80% in some parts of Asia (4). Compared with Caucasians, Indians with T2DM have low BMI and tend to be diagnosed at a younger age (5, 6). Studies from the US have found a higher incidence of diabetes among South Asians when compared with Pima Indians (7) and white Americans (8) in both BMI groups (i.e., <25 and ≥30 kg/m^2^). BMI screening as a tool for health-related conditions has limitations as it does not differentiate between lean body mass and fat mass: people with normal BMI may be adipose (total fat) or centrally obese (9). Indians are more vulnerable to T2DM than western populations due to their thin–fat phenotype and excess adiposity for a given BMI (10–12). Type 2 diabetes is heterogeneous due to its distinct pathophysiology and clinical presentations. T2DM with insulin resistance and hyperinsulinemia in individuals with obesity has been extensively studied. However, the pathophysiological mechanisms of T2DM in non-obese individuals with poor insulin secretion and poor beta-cell function are not well defined. Although several subtypes of T2DM have been identified indicating its heterogeneity (13), clinically, it is still treated as a homogeneous condition.

BKL Walawalkar Hospital, situated near the village of Dervan in Ratnagiri district of the western Indian state of Maharashtra, was established in 1996. Since its inception, it has been actively promoting rural healthcare through community outreach and education. The hospital also serves as a tertiary care referral center. It serves two coastal districts: Sindhudurg and Ratnagiri. The area has witnessed life course undernutrition from birth to adulthood (14–16). In the region (17), two distinct pathophysiologies of prediabetes among teenage girls have recently been found. This sparked our interest in examining the phenotypic, biochemical, and clinical parameters of adults with T2DM who visited the outpatient clinic of our hospital.

## Methods

### Patient recruitment

Subjects with T2DM who visited the diabetic clinic of the hospital were recruited for the study. They underwent various examinations, including demographic information, anthropometry, body composition, and laboratory measurements.

### Demographic data

Data on education, marital status, religion, occupation, and family history of diabetes were recorded at the time of registration. Additional details about history of addiction, including, smoking, tobacco chewing, and alcohol consumption, were also recorded.

### Anthropometry and body composition

The weight, height, and waist circumference were measured using standardized protocols. Body composition was measured using a bio-impedance analyzer (MC-780; Tanita Corporation, Tokyo, Japan). This analyzer does not estimate visceral fat, but gives visceral fat rating.

### Laboratory measurements

Blood samples were collected in the morning after an overnight fast. The samples were centrifuged (4°C, 3,000 rpm, 15 min) within 1 h of collection and stored at −80°C for subsequent investigations. The plasma blood glucose was measured on the ERBA 200 immediately after collection. The intra- and inter-batch coefficients of variation (CVs) were <5%. HbA_1C_ was measured using high-performance liquid chromatography (Bio-Rad D10; Bio-Rad Laboratories, Hercules, CA, USA), which was calibrated against the National Glycosylated Standardization Program with a CV of 2.8%. Fasting C-peptide was measured on an Abbott Architect i1000SR with a CV of 2.0%. The GAD65 autoantibody was measured with ELISA using DRG kits (DRG Diagnostics GmbH, Marburg, Germany) with a CV of 2.9%.

### Calculations and classifications

Three categories of BMI were created: underweight, normal weight, and overweight/obese. These refer to those with a BMI <18.5, 18.5–24.9, and ≥25 kg/m^2^, respectively (18). Non-obese denotes those who are underweight or of normal weight. Subjects with waist circumference values ≥90 cm (for men) and ≥80 cm (for women) were categorized as centrally obese (19). Those who had body fat values ≥25% (for men) and ≥35% (for women) were considered adipose (20). Using BMI and body composition, six categorical exposure groups were generated. These were as follows:

Group 1 (referred to as lean): underweight, with absence of central obesity and adiposity.

Group 2 (referred to as metabolically non-obese): normal weight, with absence of central obesity and adiposity.

Group 3: normal weight, with central obesity, but no adiposity.

Group 4: normal weight, no central obesity, but with adiposity.

Group 5: normal weight, with central obesity and adiposity.

Group 6: overweight/obese, centrally obese and adipose.

Groups 3, 4, and 5 together represent those who are metabolically obese. The glycemic indices of insulin resistance (HOMA-IR), insulin sensitivity (HOMA-S), and beta-cell function (HOMA-β) were estimated using the homeostasis model (21).

### Statistical methods

Data are presented using the median (25th–75th percentiles) for continuous variables and percentages for categorical variables. Continuous variables were assessed for normality using the Kolmogorov–Smirnov test. Grouped comparisons for continuous variables were performed using the *t*-test or ANOVA for the normally distributed variables and using the Mann–Whitney *U* test or the Kruskal–Wallis test for those non-normally distributed. Bonferroni correction for multiple comparisons was applied. Categorical associations were examined using the chi-square test. Univariate exposure refers to the BMI categories or to central obesity or adiposity. Multivariate exposures refer to the combinations of univariate exposures. The C-peptide and glycemic indices were treated as the outcomes. For the association analysis, the continuous outcomes were converted into sex-specific quartiles. Outcomes refer to membership into the lowest quartile Q_1_ for C-peptide and HOMA-β and the upper quartile Q_4_ for HOMA-IR and HOMA-S, representing low and high levels of the respective outcomes. Logistic regression was used to analyze the associations between the categorical exposures and the categorical outcomes. Odds ratios (ORs) with 95% confidence intervals (CIs) were calculated. The statistical software SPSS v25.0 and STATA 13.0 were used for the analysis.

### Ethics

This study was approved by the Institute Ethics Committee of BKL Walawalkar Rural Medical College and Hospital. Our institute’s ethics committee is registered with the Department of Health Research (DHR), Government of India (registration no. EC/NEW/INST/2023/MH/0361). Written informed consent was obtained from the subjects.

## Results

The diabetic clinic of the hospital received 757 subjects with T2DM from September 2023 to July 2024. Subjects with GAD65 autoimmune antibodies (*n* = 6) and those who were not fasting (*n* = 238) were excluded. We further excluded five subjects for whom the HOMA model failed. The final study sample included 508 eligible participants.

### Demographic characteristics

The demographic characteristics of the study population are shown in **Supplementary Table S1**.

The subjects comprised 286 (56.3%) men and 222 (43.7%) women. Of them, 86.4% had at least a secondary school education and 95.4% were married. In terms of religion, approximately 90.7% were Hindu, 4.9% Muslim, and 4.0% were Buddhist. Approximately 21% were farmers and 33% were housewives. Smoking was reported in 4%, 26% were tobacco chewers, and 26% reported alcohol consumption. A first-degree family history of diabetes was reported by 36.9%.

### Basic characteristics

The basic characteristics of the participants are displayed in **Supplementary Table S2**.

The overall median age, age at diagnosis, and duration of T2DM were 59, 51.5, and 5.2 years, respectively. Based on BMI, 52% were classified as non-obese (6% underweight and approximately 46% normal weight) and 48% as overweight/obese. Central obesity and adiposity were observed in 70% and 55.5%, respectively. The women were shorter in height, lighter in weight, and with lower waist circumference and visceral fat rating compared with the men (*p* < 0.001 for all). They also had higher BMI and body fat percentage (*p* < 0.001 for both). There were no gender differences among the glycemic parameters.

### Anthropometry, body composition, and glycemic parameters across BMI

**Table 1** shows the anthropometry, body composition, and glycemic parameters across BMI groups. The proportions of central obesity and adiposity among the non-obese were 122 (46%) and 70 (26%), respectively (data not shown). For those who were overweight, the figures were much higher, with 234 (96.3%) having central obesity and 212 (87.2%) having excess adiposity.

**Table 1:** Anthropometric, body composition, and glycemic parameters according to the BMI categories (*n* = 508).

Characteristics	Underweight	Normal weight	Overweight	p
*n*	30 (5.9%)	235 (46.3%)	243 (47.8%)	
Anthropometry	Non-obese: 265 (52.2%)		Obese: 243 (47.8%)	
Height (cm)	156.3 (148.5–168.9)	158.6 (151.8–165.0)	157.0 (151.0–164.2)	0.556
Weight (kg)	40.6 (35.8–45.8)	56.6 (50.7–62.3)	69.5 (63.3–75.9)	0.000*
		U***	U***, N***	
BMI (kg/m^2^)	16.7 ± 1.5	22.5 ± 1.7	28.4 ± 2.8	–
Waist circumference (cm)	67.9 (62.9–74.3)	86.6 (81.5–92.4)	98.5 (92.5–104.1)	0.000*
		U***	U***, N***	
Centrally obese	0	122 (51.9%)	234 (96.3%)	0.000*
Body composition				
Body fat %	15.3 (10.2–23.8)	25.0 (20.9–32.4)	36.6 (28.8–42.5)	0.000*
		U***	U***, N***	
Adipose (body fat ≥25% for men and ≥35% for women)	1 (3.3%)	69 (29.4%)	212 (87.2%)	0.000*
Visceral fat rating	3.0 (1.0–5.0)	10.0 (7.0–12.0)	13.0 (10.0–16.0)	0.000*
		U***	U***, N***	
Glycemic parameters				
Fasting glucose (mmol/L)	8.7 (6.7–13.2)	8.4 (6.5–11.2)	8.4 (6.7–11.0)	0.804
HbA_1c_ (%)	8.0 (6.3–11.1)	8.2 (6.6–10.1)	8.0 (6.9–9.7)	0.995
HbA_1c_ (mmol/mol)	64.0 (45.0–98.0)	66.0 (49.0–87.0)	64.0 (52.0–83.0)	–
Fasting C-peptide (pmol/L)	463.5 (264.9–761.5)	860.8 (629.1–1092.6)	1026.4 (827.7–1390.6)	0.000*
		U***	U***, N***	
HOMA-β	31.1 (20.5–58.9)	60.4 (35.2–98.0)	68.9 (41.2–104.5)	0.000*
		U**	U***	
HOMA-S	76.8 (52.6–106.7)	43.8 (31.8–58.7)	36.0 (26.2–45.6)	0.000*
		U***	U***, N***	
HOMA-IR	1.3 (0.9–1.9)	2.3 (1.7–3.1)	2.8 (2.2–3.8)	0.000*
		U***	U***, N***	

Data are the median (25th–75th percentiles) for continuous variables, except for BMI, for which data are the mean ± standard deviation; otherwise, *n* (%).

*p < 0.05; **p < 0.01; ***p < 0.001 (U: different from underweight; N: different from normal weight).

All of the anthropometric measurements, except for height and the body composition parameters, significantly increased as the BMI category progressed from underweight to overweight, with a statistically significant *p*-value (*p* < 0.001) for every parameter measured (**Table 1**). Among the glycemic parameters, the fasting glucose and HbA_1C_ concentrations were similar in all groups. The fasting C-peptide concentrations, HOMA-β, and HOMA-IR statistically increased, while HOMA-S decreased from the lean to the overweight group (*p* < 0.001 for all).

**Table 2** displays glycemic parameters between centrally obese and non-obese, adipose and non-adipose. The fasting C-peptide concentrations, HOMA-β, and HOMA-IR were higher, while HOMA-S was lower, in those who were centrally obese (*p* < 0.001 for all indices). Similar results were obtained for subjects with adiposity (*p* < 0.05 for HOMA-β and *p* < 0.001 for HOMA-S and HOMA-IR).

**Table 2:** Central obesity, adiposity, and glycemic parameters.

Characteristics	Centrally obese	Centrally non-obese	P	Adipose	Non-adipose	P
*n*	356 (70.1%)	152 (29.9%)		282 (55.5%)	226 (44.5%)	
Fasting glucose (mmol/L)	8.1 (6.5–11.0)	9.1 (6.9–11.2)	0.062	8.5 (6.6–11.1)	8.2 (6.6–10.8)	0.603
HbA_1c_ (%)	8.0 (6.8–9.7)	8.4 (6.7–10.6)	0.107	8.0 (6.8–10.0)	8.1 (6.6–9.9)	0.945
HbA_1c_ (mmol/mol)	64.0 (51.0–83.0)	68.0 (50.0–92.0)	–	64.0 (51.0–86.0)	65.0 (49.0–85.0)	–
Fasting C-peptide (pmol/L)	960.2 (761.5–1291.2)	761.5 (529.7–960.2)	0.000***	993.3 (761.3–1324.0)	827.5 (595.8–1059.2)	0.000***
HOMA-β	69.1 (40.9–106.1)	46.8 (28.2–79.8)	0.000***	66.4 (39.8–104.3)	58.4 (32.9–93.9)	0.033*
HOMA-S	38.0 (28.2–49.6)	48.1 (33.7–66.8)	0.000***	36.4 (27.7–48.3)	45.8 (34.1–63.6)	0.000***
HOMA-IR	2.6 (2.0–3.5)	2.1 (1.5–2.9)	0.000***	2.7 (2.1–3.6)	2.2 (1.6–2.9)	0.000***

Data are the median (25th–75th percentiles); otherwise, *n* (%).

*p < 0.05; ***p < 0.001.

### Body composition exposure and glycemic outcome

The multiple body composition exposures and outcomes are shown in **Table 3**.

**Table 3:** Multiple body composition exposures and glycemic outcomes.

Characteristics	Group 1 (lean) (*n* = 29)	Group 2 (*n* = 98)	Group 3 (*n* = 68)	Group 4 (*n* = 15)	Group 5 (*n* = 54)	Group 6 (*n* = 204)
Underweight (yes/no)	Yes	No	No	No	No	No
Normal weight (Yes/No)	No	Yes	Yes	Yes	Yes	No
Overweight/obese (Yes/No)	No	No	No	No	No	Yes
Central obesity (Yes/No)	No	No	Yes	No	Yes	Yes
Adiposity (Yes/No)	No	No	No	Yes	Yes	Yes
Gender (Men/Women)	(18/11)	(72/26)	(39/29)	(9/6)	(26/28)	(93/111)
BMI (kg/m^2^)	16.7 ± 1.5	21.3 ± 1.6	23.1 ± 1.4	22.3 ± 1.6	23.8 ± 0.9	28.7 ± 2.7
Visceral fat rating	3.0 (1.0–5.0)	8.0 (6.0–11.0)	10.0 (7.0–12.0)	9.0 (6.0–12.0)	10.0 (8.75–14.0)	13.0 (10.0–16.0)
		1**	1***	1*	1***,2**	(1–3)***,4**,5*
Fasting glucose (mmol/L)	9.0 (6.6–13.4)	9.0 (7.0–11.0)	7.5 (5.9–10.5)	9.5 (5.9–17.8)	8.7 (6.4–11.4)	8.5 (6.7–11.1)
HbA_1c_ (%)	8.0 (6.4–11.2)	8.4 (6.8–10.4)	7.8 (6.5–9.4)	7.6 (5.5–11.5)	8.5 (6.4–10.2)	7.9 (6.9–9.7)
HbA_1c_ (mmol/mol)	64.0 (46.0–99.0)	68.0 (51.0–90.0)	62.0 (48.0–79.0)	60.0 (37.0–102.0)	69.0 (46.0–88.0)	63.0 (52.0–83.0)
Fasting C-peptide (pmol/L)	463.4 (264.8–761.3)	794.4 (562.7–1059.2)	926.8 (628.9–1191.6)	860.6 (595.8–1456.4)	827.5 (695.1–1158.5)	1059.2 (794.4–1423.3)
		1**	1***	1*	1***	1***,2***,3*,5*
HOMA-β	29.8 (20.4–60.2)	50.2 (34.2–81.9)	71.2 (41.7–114.2)	65.8 (28.0–112.1)	59.2 (36.8–98.1)	71.6 (40.9–105.0)
			1***			1***,2*
HOMA-S	77.0 (53.4–107.0)	44.5 (33.6–62.9)	44.7 (34.1–60.8)	32.3 (23.2–50.2)	43.2 (31.7–52.8)	35.5 (25.8–45.4)
		1**	1***	1***	1***	1***,2***,3**
HOMA-IR	1.3 (0.9–1.9)	2.2 (1.6–2.9)	2.2 (1.6–2.9)	3.1 (2.0–4.3)	2.3 (1.9–3.1)	2.8 (2.2–3.9)
		1**	1***	1***	1***	1***,2***,3**

Data are the median (25th–75th percentiles) for continuous variables, except for BMI, for which data are the mean ± standard deviation.

*Y*, yes; *N*, no.

*p < 0.05; **p < 0.01; ***p < 0.001.

The fasting glucose and HbA_1C_ levels were statistically similar in all groups. However, C-peptide and HOMA-IR were significantly lower and HOMA-S significantly higher in the lean group (*p* < 0.05 for all). The HOMA-β of the lean group was different from those of groups 3 and 6 (*p* < 0.001 for both).

**Figure 1:**
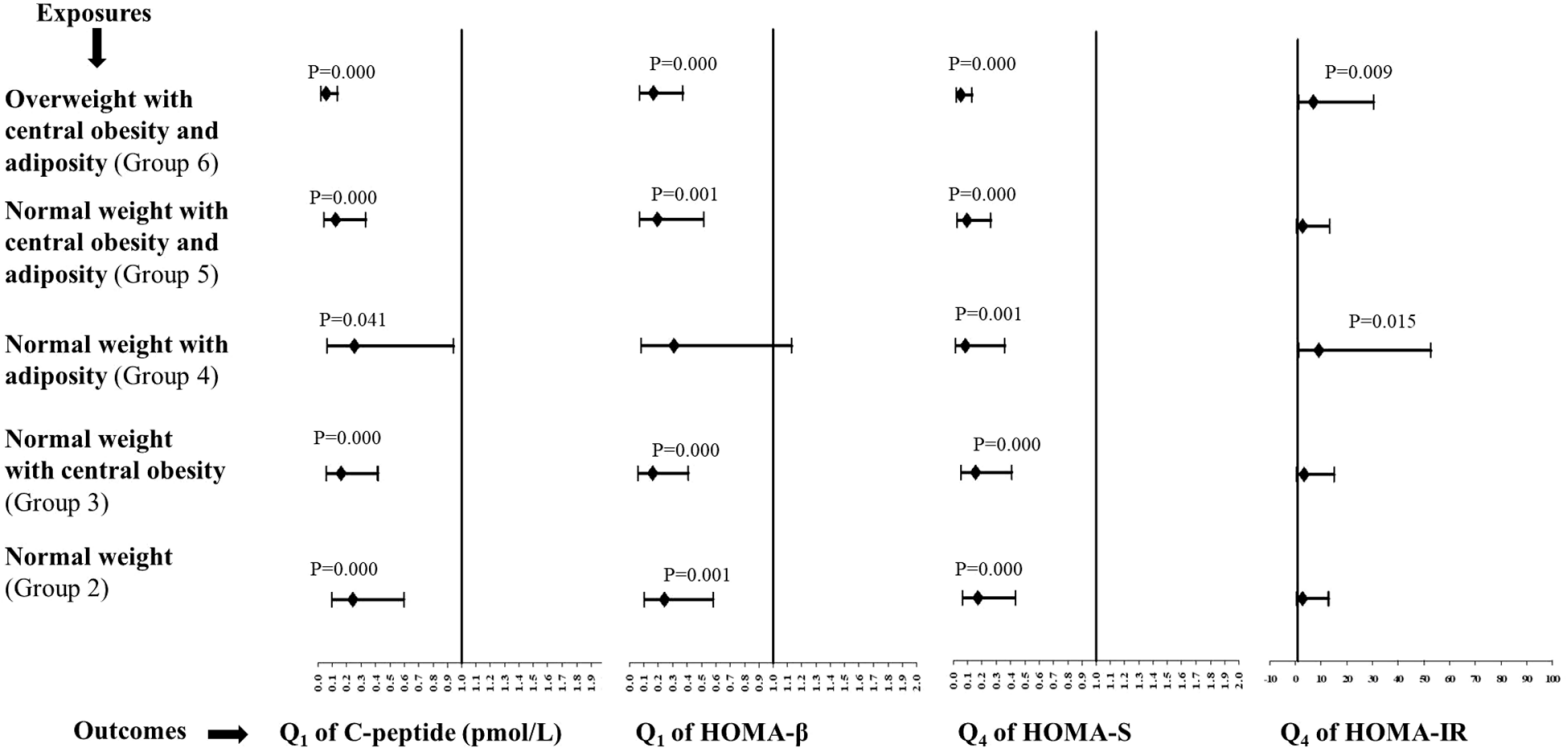
Multivariate odds ratios (95% confidence intervals) for all groups in relation to group 1 (the lean group). Sex-specific quartiles were calculated for C-peptide, HOMA-β, HOMA-S, and HOMA-IR. *Q1* and *Q4* are the first and fourth quartiles of the metabolic outcomes, respectively.

The multivariate exposures and categorical risk outcomes are demonstrated in **Figure 1**.

The likelihood of a low C-peptide was significantly reduced in groups 2–6 compared with the lean group. The ORs (95%CIs) for groups 2, 3, 4, 5, and 6 were 0.24 (0.1–0.6), 0.16 (0.06–0.42), 0.25 (0.07–0.94), 0.12 (0.04–0.34), and 0.05 (0.02–0.14), respectively. The ORs (95%CIs) for poor beta-cell function were 0.24 (0.10–0.58) for group 2, 0.16 (0.06–0.41) for group 3, 0.19 (0.07–0.51) for group 5, and 0.16 (0.07–0.37) for group 6, which were significantly reduced in comparison to the lean group. The ORs for high insulin sensitivity were significantly reduced in all the groups, from group 2 to 6, in comparison to the lean group. The ORs (95%CIs) were 0.17 (0.07–0.43) for group 2, 0.15 (0.06–0.41) for group 3, 0.08 (0.02–0.36) for group 4, 0.09 (0.03–0.26) for group 5, and 0.05 (0.02–0.13) for group 6. There was a significant likelihood of an elevated insulin resistance observed for groups 4 and 6 in comparison to the lean group. The ORs (95%CIs) for groups 4 and 6 were 9.0 (1.5–52.8) and 7.0 (1.6–30.5), respectively.

A multivariate analysis was also separately conducted for men and women (**Supplementary Figure S1** and **Supplementary Figure S2** for men and women, respectively). The likelihood of a low C-peptide was significantly reduced in groups 2–6, except for group 4, in relation to the lean group for both sexes. The ORs for poor beta-cell function were significantly lower than those for the lean group in all groups, except for group 4, for both sexes. The ORs for high insulin sensitivity were significantly reduced in all groups, from group 2 to 6, in relation to the lean group for both sexes. The results for elevated insulin resistance in relation to the lean group were exactly similar for men. There was no association of elevated insulin resistance with different groups in women.

## Discussion

As expected, all of the anthropometric and body composition parameters increased across BMI categories. Among the glycemic parameters, glucose and HbA_1C_ were similar. C-peptide, HOMA-β, and HOMA-IR showed an increasing trend, while that for HOMA-S was decreasing. The underweight from the non-obese group did not have central obesity and adiposity compared with those with normal weight, who had substantial central obesity and adiposity, highlighting the role of adiposity rather than BMI, as suggested by Das (4). Group 1, which comprised lean participants, had the lowest C-peptide and insulin resistance, while group 2, comprising those metabolically non-obese, had higher visceral fat rating, C-peptide, and insulin resistance than those in group 1. The subjects in groups 3, 4, and 5 (those with normal BMI with either central obesity or adiposity or both), representing the metabolically obese, had higher C-peptide and insulin resistance compared with those in the lean group. This pathophysiology is similar to that of insulin resistance observed in obese T2DM, as suggested by Scott et al. al (22). This can be attributed to the increased visceral fat, which increases the release of free fatty acids, causing insulin resistance (23). Subjects from group 6 (overweight, centrally obese, and adipose) showed glycemic characteristics similar to those observed in classical T2DM. The role of adiposity was once again confirmed by the fact that those in groups 4 and 6 had significantly higher ORs for high HOMA-IR than the lean group. Thus, our results highlighted the heterogeneity of T2DM.

Characterization of T2DM by identifying various subtypes has been attempted across various parts of the world (13) and India (24–26). Subtyping has been performed using a cluster analysis approach. Most of these approaches have used BMI as one of the clustering variables (24–26). However, there are several drawbacks of BMI as it does not reflect muscle mass or gender differences (27). It is also unable to represent the total and regional adiposity (subcutaneous and visceral) (23) and ectopic fat (28), which are known to play a role in the pathophysiology of T2DM. The role of body composition has not been taken into account in any of the reports that characterized T2DM using cluster analysis. In this study, we used simple anthropometry, body composition, and glycemic parameters to characterize the lean group. Most of the characteristics (the lowest BMI and waist circumference, as well as the lowest C-peptide levels, HOMA-β, and HOMA-IR) of our lean group resonated with those of a subtype termed severely insulin-deficient diabetes (SIDD), described by Mohan et al. (25) and Tripathi et al. (26). The SIDD subtype described by Mohan et al. has a wide spectrum of BMI (mean, 24.9 kg/m^2^) (29), while that described by Tripathi et al. (26) has a median BMI of 24.5 kg/m^2^. A similar report by Prasad et al. (24) showed a mean BMI of 25.0 kg/m^2^ for the SIDD cluster. The median BMI for our lean group was only 17.4 kg/m^2^. A somewhat similar approach to ours was used by Lontchi-Yimagou et al. (30) to identify lean diabetes in South India, where the mean BMI was 18.3 kg/m^2^. Our lean group in comparison to that in this report had the lowest mean BMI of only 16.7 kg/m^2^. A similar phenotype having a low BMI has also been described among diabetic populations in Africa (31, 32). By categorizing those with a normal BMI (a mean BMI of 22.5 kg/m^2^) based on their body composition, we were able to distinguish them from the lean group, making our lean group a distinct identity of lean diabetes. This could be due to the life course undernutrition resulting in a small organ size with reduced metabolic capacity, leading to poor insulin secretion, resulting in T2DM, as suggested by the DOHaD (developmental origins of health and disease) hypothesis (20, 33).

The treatment regimen for T2DM has always been homogeneous despite its heterogeneity. It is glucocentric, solely based on glucose and HbA_1C_. Currently, the American Diabetes Association (ADA) guidelines (34) are used in India for the treatment of diabetes. They are based on studies predominantly performed on white Caucasians (31), who have a different phenotype from Indians. Metformin is used as a first-line therapy across India and is easily available in rural primary health centers (35). However, its use in lean diabetes with insulin secretory defects is inappropriate (36), and its impact has never been explored (31). It is also unclear whether insulin secretagogues can be used effectively in lean diabetes (30). Insulin could be the better choice for glycemic control; however, its availability and affordability are the main issues in rural areas. In addition, food insecurity and the lack of availability of point-of-care devices for glucose monitoring may aggravate hypoglycemic situations (37). The time has come to move beyond BMI and reassess the pathophysiology of T2DM among those non-obese and reconsider a one-size-fits-all treatment approach. Recently, the International Diabetes Federation (IDF) has formally classified this as type 5 diabetes and has constituted a working group to develop formal diagnostic criteria and therapeutic guidelines (38).

The major strength of our report is that it comes from a region where there have been no prior hospital- or community-based studies/reports on subjects with T2DM. We were able to highlight the heterogeneity of T2DM even with the simple measures of anthropometry and body composition, which included both obesity and adiposity. This also highlighted the incapability of BMI to reflect the true phenotype of T2DM. There are limitations to our study as well. It is a cross-sectional snapshot of subjects with T2DM at a hospital, which does not reflect the lean phenotypic presentation of the community. The subjects recruited from the hospital included some severe cases, which might have led to the overestimation of certain glycemic abnormalities. In addition, our subjects were not newly diagnosed. The variable duration of the disease may have affected the interpretation of markers such as C-peptide and the HOMA indices. Undernutrition was noted as a result of a low BMI, but there was no evaluation of the dietary or nutritional status. Our findings may not be directly applicable to other rural populations due to India’s diverse sociocultural, genetic, and environmental factors. However, replicating this simple model of body composition, which goes beyond BMI and the glycemic parameters, can be useful in identifying the regional heterogeneity of T2DM.

## Conclusion

We have demonstrated the heterogeneity of T2DM in our resource-constrained region using simple anthropometric and body composition measurements. This is a cross-sectional study performed in a tertiary care hospital, hence does not represent the general population. However, it revealed a specific lean phenotype that encompassed a low BMI, the absence of central obesity and adiposity, poor insulin secretion, and low insulin resistance. Large community-based studies are necessary to identify the subtypes. The development of precision medicine for non-obese T2DM will be a long-term goal, which will involve studying complex molecular mechanisms and conducting randomized control trials. The time has come to improve the conventional glucocentric, one-size-fits-all approach for glycemia treatment with simple body composition measurements and glycemic parameters. Our approach is simple, affordable, and applicable and can be put into practice in resource-limited settings in low- and middle-income countries (LMICs) with ease.

## Data Availability

The raw data supporting the conclusions of this article will be made available by the authors, without undue reservation and subject to permission by the Institute Ethics Committee (IEC) and the Health Ministry Screening Committee (HMSC), Government of India.
