# Supplementary material for: Heterogeneity of type 2 diabetes in rural India
Source: Front Endocrinol (Lausanne). 2025 Sep 9;16:1524194. doi: 10.3389/fendo.2025.1524194 (PMC12454038; doi:10.3389/fendo.2025.1524194)

**Supplementary Table 1: Demographic characteristics of the patients (n=508)**

| **Characteristics** | **n (%)** |
| --- | --- |
| **Gender** |  |
| Male | 286 (56.3) |
| Female | 222 (43.7) |
| **Education** |  |
| Educated (at least matriculate) | 439 (86.4) |
| **Marital status** |  |
| Married | 480 (94.5) |
| Unmarried | 6 (1.2) |
| Divorce | 5 (1.0) |
| Widow | 17 (3.3) |
| **Religion** |  |
| Hindu | 461 (90.7) |
| Muslim | 25 (4.9) |
| Buddha | 20 (4.0) |
| Jain | 2 (0.4) |
| **Occupation** |  |
| Farming | 106 (20.8) |
| Petty Business | 2 (0.4) |
| Business self | 20 (4.0) |
| Service professional | 26 (5.1) |
| Service non-professional | 7 (1.4) |
| Skilled worker | 35 (6.9) |
| Unskilled worker | 30 (5.9) |
| Transport | 4 (0.8) |
| Unemployed | 7 (1.4) |
| Maid | 2 (0.3) |
| Housewife | 170 (33.5) |
| Pensioner | 18 (3.6) |
| Other | 81 (15.9) |
|  |  |
| **Substance abuse** |  |
| Smoker | 20 (4.0) |
| Tobacco chew | 132 (26.0) |
| Alcohol | 76 (15.0) |
|  |  |
| **Family history of Diabetes (1^st^ degree)** |  |
| Yes | 187 (36.9) |
| No | 267 (52.5) |
| Don’t know | 54 (10.6) |

Data are n (%)

**Supplementary Table 2: Basic characteristics of diabetic subjects**

| **Characteristics** | **All** | **Male** | **Female** | **P** |
| --- | --- | --- | --- | --- |
|  | (n=508) | 286 (56.3%) | 222 (43.7%) |  |
| Age (y) | 58.9 (51.6-67.0) | 59.9 (53.1-68.5) | 57.7 (50.0-65.1) | 0.012* |
| Age at diagnosis of DM (y) | 51.5 (44.0-59.1) | 52.6 (44.5-60.4) | 50.6 (43.2-57.8) | 0.083 |
| Duration of DM (y) | 5.2 (2.0-10.8) | 5.2 (2.1-11.0) | 5.6 (1.6-10.4) | 0.468 |
| **Anthropometry** |  |  |  |  |
| Height (cm) | 158.1 (151.4-164.7) | 163.3 (159.3-168.1) | 151.0 (146.9 -155.0) | 0.000* |
| Weight (kg) | 62.2 (54.0-70.2) | 65.5 (57.9-72.1) | 58.6 (50.8-66.2) | 0.000* |
| BMI (kg/m^2^) | 24.7 (22.2-27.5) | 24.4 (22.1-26.6) | 25.5 (22.5-28.8) | 0.000* |
| Underweight | 30 (5.9%) | 19 (6.6%) | 11 (5.0%) |  |
| Normal Weight | 235 (46.3%) | 146 (51.0%) | 89 (40.1%) | 0.018* |
| Overweight | 243 (47.8%) | 121 (42.3%) | 122 (55.0%) |  |
| Waist circumference (cm) | 92.0 (84.4-98.5) | 92.6 (86.1-99.5) | 88.9 (81.3-97.8) | 0.001* |
| Centrally Obese |  |  |  |  |
| Yes | 356 (70.1%) | 181 (63.3%) | 175 (78.8%) | 0.000* |
| No | 152 (29.9%) | 105 (36.7%) | 47 (21.2%) |  |
| **Body composition** |  |  |  |  |
| Body fat % | 29.5 (23.3-37.7) | 24.3 (20.6-28.9) | 37.8 (33.3-42.8) | 0.000* |
| Adiposity |  |  |  |  |
| Yes | 282 (55.5%) | 134 (46.9%) | 148 (66.7%) | 0.000* |
| No | 226 (44.5%) | 152 (53.1%) | 74 (33.3%) |  |
| Visceral fat rating | 11.0 (8.0-14.0) | 13.0 (10.0-15.0) | 8.0 (6.0-11.0) | 0.000* |
| **Glycaemic Parameters** |  |  |  |  |
| Fasting glucose (mmol/L) | 8.4 (6.6-11.0) | 8.2 (6.8-11.0) | 8.5 (6.5-11.1) | 0.790 |
| HbA_1c_ (%) | 8.1 (6.8-9.9) | 8.2 (6.8-10.0) | 7.9 (6.6-9.9) | 0.254 |
| HbA_1c_ (mmol/mol) | 65.0 (51.0-85.0) | 66.0 (51.0-86.0) | 63.0 (49.0-85.0) | - |
| Fasting C-peptide (pmol/L) | 926.8 (695.1-1257.8) | 926.8 (662.0-1290.9) | 893.7 (695.1-1158.5) | 0.506 |
| HOMA-β | 61.3 (36.0-100.0) | 61.0 (35.5-97.3) | 61.5 (36.7-104.3) | 0.977 |
| HOMA-S | 40.7 (29.4-53.3) | 40.2 (28.4-53.4) | 41.6 (30.8-53.4) | 0.408 |
| HOMA-IR | 2.4 (1.9-3.4) | 2.5 (1.9-3.5) | 2.4 (1.9-3.2) | 0.408 |

Data are median (25th -75^th^ percentiles) or n (%)

**Supplementary Figure 1: Odds ratio (95% confidence intervals) for all the groups in relation to Group 1 (lean) for males**


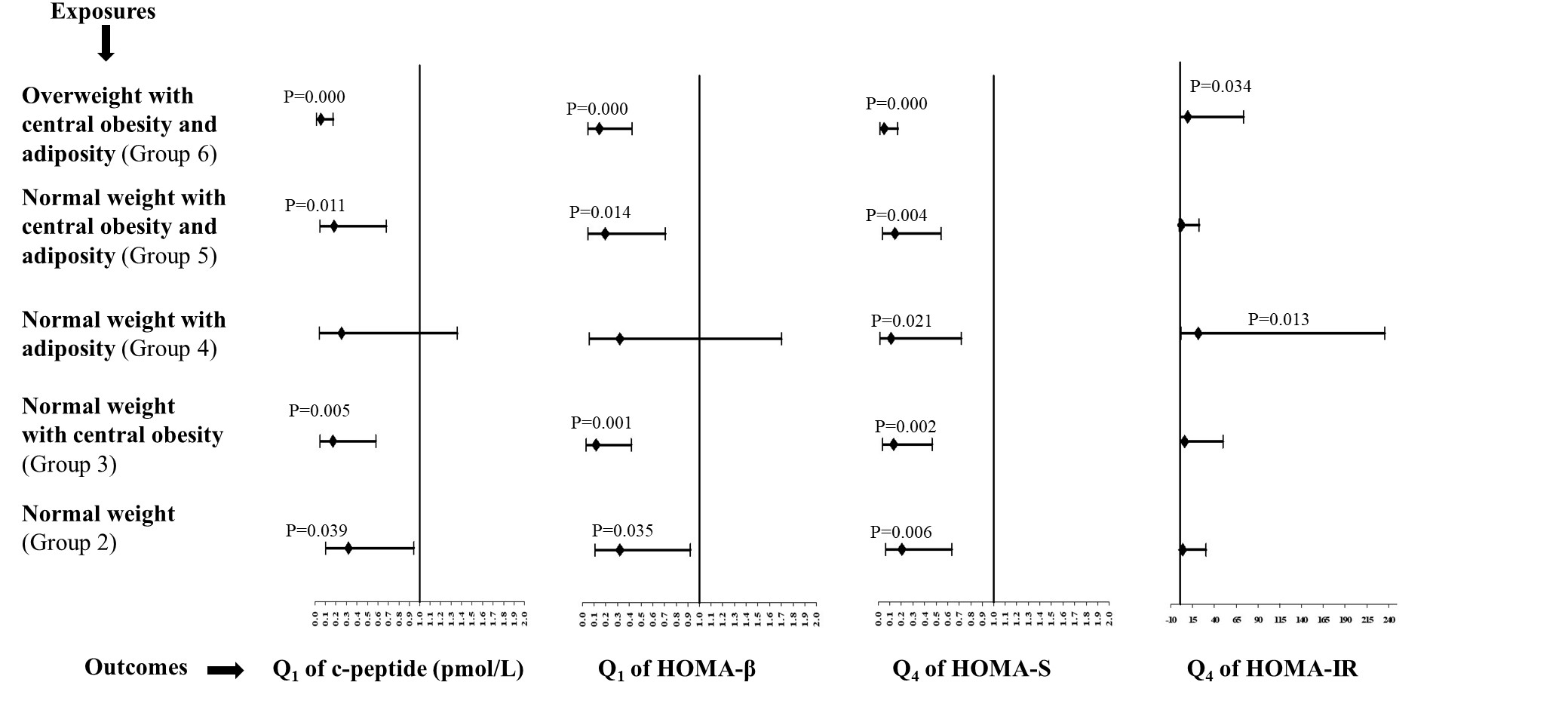


**Supplementary Figure 2: Odds ratio (95% confidence intervals) for all the groups in relation to Group 1 (lean) for females**


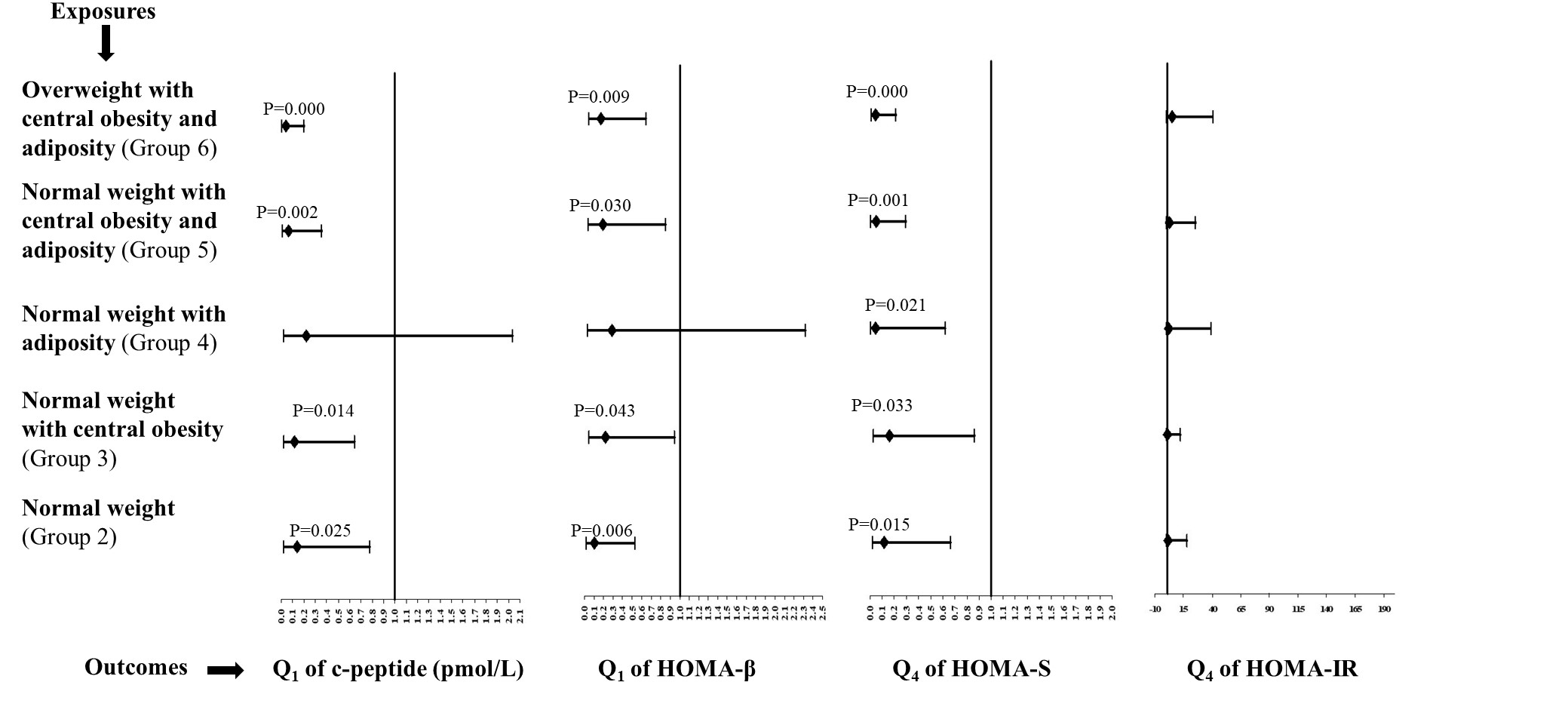

Supplement: Supplementary file 1 [file DataSheet1.docx]
